# TM9SF1 offers utility as an efficient predictor of clinical severity and mortality among acute respiratory distress syndrome patients

**DOI:** 10.3389/fimmu.2024.1408406

**Published:** 2024-06-03

**Authors:** Fengsheng Cao, Lu Zhang, Zhenwang Zhao, Xiaofang Shen, Jinsong Xiong, Zean Yang, Baoxian Gong, Mingming Liu, Huabo Chen, Hong Xiao, Min Huang, Yang Liu, Guangyu Qiu, Ke Wang, Fengqiao Zhou, Juan Xiao

**Affiliations:** ^1^ Department of Critical Care Medicine & Department of Emergency Medicine, Xiangyang Central Hospital, Affiliated Hospital of Hubei University of Arts and Science, Xiangyang, Hubei, China; ^2^ Medical College, Hubei University of Arts and Science, Xiangyang, Hubei, China; ^3^ Institute of Neuroscience and Brain Diseases, Xiangyang Central Hospital, Affiliated Hospital of Hubei University of Arts and Science, Xiangyang, Hubei, China; ^4^ Gucheng People’s Hospital, Affiliated Hospital of Hubei University of Arts and Science, Xiangyang, Hubei, China

**Keywords:** ARDS, TM9SF1, prediction model, severity, mortality

## Abstract

**Introduction:**

Acute respiratory distress syndrome (ARDS) is a major cause of death among critically ill patients in intensive care settings, underscoring the need to identify biomarkers capable of predicting ARDS patient clinical status and prognosis at an early time point. This study specifically sought to explore the utility and clinical relevance of *TM9SF1* as a biomarker for the early prediction of disease severity and prognostic outcomes in patients with ARDS.

**Methods:**

This study enrolled 123 patients with severe ARDS and 116 patients with non-severe ARDS for whom follow-up information was available. The mRNA levels of *TM9SF1* and cytokines in peripheral blood mononuclear cells from these patients were evaluated by qPCR. The predictive performance of *TM9SF1* and other clinical indicators was evaluated using received operating characteristic (ROC) curves. A predictive nomogram was developed based on *TM9SF1* expression and evaluated for its ability in the early prediction of severe disease and mortality in patients with ARDS.

**Results:**

*TM9SF1* mRNA expression was found to be significantly increased in patients with severe ARDS relative to those with non-severe disease or healthy controls. ARDS severity increased in correspondence with the level of *TM9SF1* expression (odds ratio [OR] = 2.43, 95% confidence interval [CI] = 2.15–3.72, *P* = 0.005), and high *TM9SF1* levels were associated with a greater risk of mortality (hazard ratio [HR] = 2.27, 95% CI = 2.20–4.39, *P* = 0.001). ROC curves demonstrated that relative to other clinical indicators, *TM9SF1* offered superior performance in the prediction of ARDS severity and mortality. A novel nomogram incorporating *TM9SF1* expression together with age, D-dimer levels, and C-reactive protein (CRP) levels was developed and was used to predict ARDS severity (AUC = 0.887, 95% CI = 0.715–0.943). A separate model incorporating *TM9SF1* expression, age, neutrophil-lymphocyte ratio (NLR), and D-dimer levels (C-index = 0.890, 95% CI = 0.627–0.957) was also developed for predicting mortality.

**Conclusion:**

Increases in ARDS severity and patient mortality were observed with rising levels of *TM9SF1* expression. *TM9SF1* may thus offer utility as a novel biomarker for the early prediction of ARDS patient disease status and clinical outcomes.

## 1 Introduction

Acute respiratory distress syndrome (ARDS) is a serious clinical condition wherein extensive alveolar capillary injury occurs following pulmonary insults such as shock, trauma, or severe infection, resulting in characteristic symptoms including tachypnea, distress, and progressive hypoxemia together with diffuse alveolar infiltration evident on X-ray imaging (1, 2). While there have been many improvements to the management of ARDS in patients over recent years such as the advent of strategies for mechanical ventilation that protect lung integrity (3, 4), prone positioning, and extracorporeal membrane oxygenation (5), adequately treating ARDS patients in intensive care unit (ICU) settings remains a pressing challenge. ARDS affects a large percentage of critical patients admitted to the ICU (6), culminating in a mortality rate as high as 35% among these severely ill individuals (3). In recent reports, disease severity was found to be strongly dependent on a range of host factors, underscoring the importance of fully characterizing these individual responses at a molecular level (7, 8). While severe genes reportedly linked with ARDS progression have been identified to date, the predictive performance as tools for evaluating disease prognosis and severity remains unsatisfactory. These factors thus hamper clinical efforts to predict the deterioration of patient vital status during the earlier stages of disease. There is thus a critical need for the investigation of individualized biomarkers capable of predicting ARDS patient status and clinical outcomes.

At the tissue level, ARDS pathophysiology is characterized by diffuse alveolar epithelial cell apoptosis or necrosis as a result of exposure to pathogens or noninfectious irritants (9, 10), with the clinical status of affected patients thus being profoundly shaped by multiple proinflammatory mechanisms. ARDS and associated progression to the development of multi-organ failure (MOF) have been proposed as forms of secondary bystander tissue damage that lie along a spectrum and stem from the effects of dysregulated or excessive inflammatory responses to a primary infectious or non-infectious insult (11). Factors associated with the regulation of such inflammation thus hold great promise as potential biomarkers that may be analyzed to more reliably assess ARDS severity and the prognosis of affected patients.

In a previous study, transmembrane 9 superfamily member 1 (TM9SF1) was identified as a key player in the processes that shape inflammatory lung injury (12). TM9SF1 is a TM9SF family protein with 9 transmembrane domains and a large non-cytoplasmic region, and its expression at the mRNA level is altered in a mouse model of lipopolysaccharide (LPS)-induced acute lung injury (12). Mice in which *TM9SF1* has been knocked out also presented with significant changes in lung injury progression, including altered inflammatory cytokine expression and infiltration by inflammatory cells. Given these prior results linking the expression of *TM9SF1* to immune regulation and inflammatory lung injury progression, this suggested the possibility that changes in *TM9SF1* levels may play a role in ARDS incidence and the development of additional severe lung injury as a consequence of its regulatory effects on immune-mediated inflammatory activity.

In this study, a prospective cohort analysis was conducted enrolling both severe and non-severe ARDS patients admitted to Xiangyang Central Hospital, as well as healthy control (HC) subjects collected during this same period of time. These analyses were designed to clarify the differences in *TM9SF1* expression in ARDS patients, to better probe the clinical relevance and possible functional role of *TM9SF1* as a novel personalized biomarker for the early-stage prediction of ARDS severity and patient prognosis, and to construct predictive models capable of the early identification of patients with severe disease and gauging their prognosis.

## 2 Materials and methods

### 2.1 Study population

This was a prospective cohort study of adult ARDS patients admitted to the medical floor or the medical ICU of Xiangyang Central Hospital from November 1, 2022 through January 31, 2023, with follow-up having been performed through March 31, 2023. Patients were considered eligible for the study if they had been admitted to the hospital for symptoms consistent with ARDS caused by pneumonia, aspiration, trauma, and sepsis, among other conditions. ARDS was diagnosed as per the Berlin Definition.

ARDS disease severity was used to classify patients into severe and nonsevere groups (13). Patients were classified in the severe group if they were admitted to the ICU and exhibited any: (1) respiratory failure, as characterized by a respiratory rate ≥ 30 breaths/min and resting arterial oxygen saturation ≤ 93% mainly for patients without invasive mechanical ventilation; and/or (2) an arterial blood oxygen partial pressure/fraction of inspiration O_2_ (PaO_2_/FiO_2_) ≤ 150 mmHg mainly for those with invasive mechanical ventilation. Patients were classified in the nonsevere group if they were hospitalized and admitted to the internal medicine or pneumology wards without the need for critical care. Any ARDS patients not meeting the criteria set forth for the severe group were included as nonsevere cases.

ARDS patients were excluded from this study if they met any of the following criteria: (1) patients ≤ 18 years of age; (2) patients that died within 24 h following admission; (3) patients with a history of chronic respiratory disease (such as chronic obstructive pulmonary disease and chronic pulmonary fibrosis), immunodeficiency, or malignant tumors; (4) patients for whom severity status was not established; or (5) patients missing details regarding age, sex, or the results of key clinical laboratory tests. In addition, 52 healthy subjects who had undergone physical examinations in the Physical Examination Center and who generally matched the age and sex distributions of the enrolled ARDS patients were included in this study as HCs.

### 2.2 Clinical details and sample collection

Basic ARDS patient clinical data including demographic, clinical, and laboratory data were extracted from electronic medical records by trained research staff using standardized forms.

Laboratory results collected within 24 h of patient admission were collected for analysis, including results pertaining to the following: white blood cells (WBCs), hemoglobin (HGB), hematocrit (HCT), lymphocytes, lymphocyte % (LYM%), monocytes, monocyte % (MON%), neutrophils, neutrophil % (NEU%), platelet lymphocyte ratio (PLR), monocyte lymphocyte ratio (MLR), neutrophil-lymphocyte ratio (NLR), platelets (PLTs), platelet distribution width (PDW), mean platelet volume (MPV), C-reactive protein (CRP), procalcitonin (PCT), activated partial thromboplastin time (APTT), prothrombin time (PT), thrombin time (TT), D-dimer, international normalized ratio (INR), and erythrocyte sedimentation rate (ESR). Oxygenation status in ARDS patients was evaluated based on the PaO_2_/FiO_2_ ratio, while the numbers of organ failures on ICU admission were assessed based on Sequential Organ Failure Assessment (SOFA) scores (14).

The biochemical test results for these patients detailed above were collected through analyses conducted with an Atellica Solution Immunoassay and Clinical Chemistry Analyzer (Siemens Healthcare Diagnostics, Erlangen, Germany). WBCs and lymphocytes were estimated with a Sysmex XE-2100 (TOA Medical Electronics, Kobe, Japan), while a Sysmex CS-5100 System (Siemens Healthcare Diagnostics, Erlangen, Germany) was used to measure APTT, PT, INR, fibrinogen, and D-dimer levels. Levels of CRP were quantified with an i-CHROMA instrument (BodiTech Med Inc., Chuncheon, Korea), whereas the Vacuette ESR System (Greiner Bio-One GmbH, Frickenhausen, Germany) was used to quantify ESR values.

The blood samples analyzed for this study were obtained on enrollment from remaining blood used for routine complete blood counts in the clinic after admission to the ICU or clinical ward. Additionally, blood samples from 61 ARDS patients were obtained when patients were both experiencing severe disease and in stable remission. “Remission” was defined as successful completion of the SBT test for 120 min and with no need for re-intubation after extubation for at least 48 h or tracheotomy status after invasive mechanical ventilation without ventilator support lasting longer than 48 h.

### 2.3 Follow-up

Patients were followed from the time of hospital admission to discharge or death, with no interference with medical decision-making for the enrolled patients. The follow-up for the patients included in this study continued until March 31, 2023. The endpoint for follow-up for patients with severe ARDS was death or recovery from illness. The outcome-related information was collected for further analysis, including survival status, discharge date, days of hospitalization, and date of death date.

### 2.4 Separation of peripheral blood mononuclear cells

Blood samples remaining in the clinic after routine complete blood count (CBC) examinations were collected for the separation of PBMCs. The PBMCs were isolated from 2 mL whole blood by density centrifugation at 3000 rpm for 20 min with 3 mL of Human Lymphocyte Separation Medium (Solarbio, P8610), with collection of the PBMCs from the second layer (layers from the top to the bottom: plasma, PBMCs, separation medium, and erythrocyte layers). The PBMCs were resuspended in 1 mL of TRIzol reagent (Invitrogen, 15596026) prior to storage at – 80 °C.

### 2.5 RNA extraction

The PBMC samples in TRIzol were removed from the –80°C freezer and were thawed on ice before the addition of 200 μL of chloroform, followed by thorough mixing and incubation on ice for 15 min. The samples were then centrifuged at 12 000 rpm for 15 min at 4°C. The upper aqueous phase was transferred to another EP tube, taking care not to aspirate the interphase, and an equal volume of isopropanol was added, followed by thorough mixing and incubation on ice for 15 min. This was followed by further centrifugation (12 000 rpm, 15 min, 4°C), the supernatant was discarded, and the RNA was allowed to pellet at the bottom of the tube. After the addition of 1 mL of 75% ethanol, the pellet was resuspended with gentle shaking, and centrifuged (7500 rpm, 5 min, 4°C). The supernatant was discarded as much as possible and the pellet was air-dried at room temperature for 5–10 min. The RNA was dissolved in 40 μL of 0.1% DEPC water (adding DEPC water according to the amount of pellet) and stored at -20°C until use.

### 2.6 Reverse transcription

The RNA was reverse-transcribed to cDNA using an iScript cDNA Synthesis Kit (Bio-Rad, 1708891) according to the provided directions. The reverse transcription was performed in a reaction volume of 20 µL, including 15 μL of RNA solution together with 1000 ng RNA, 4 μL of 5x reverse-transcription reaction mix, and 1μL of iScript reverse transcriptase at 25°C for 5 min, followed by 46°C for 5 min and 95°C for 1 min. After the reaction, the cDNA was collected and stored at 4°C or -20°C.

### 2.7 qPCR

Relative gene expression levels were calculated using the 2^-ΔΔCt^ method, using GAPDH as a reference as in prior reports (15). Primer sequences used for this study are presented in **Supplementary Table S1**. qPCR was performed on the ABI7500 system by using the iTaq Universal SYBR Green Supermix (Bio-Rad, 1725122). The reaction volume was 20 µL consisting of 3.0 μL of diluted cDNA, 1.0 μL of the forward and reverse primers (10 μM), 10 μL of iTaq Universal SYBR Green Supermix (Bio-Rad, 1725122), and 5 µL of water with the following conditions: 95°C for 5 min; 40 cycles at 95°C for 10 s; 60°C for 20 s.

### 2.8 Western blotting

Western blotting was performed as previously described (16). The primary antibodies used for the reactions included anti- GAPDH (GoodHere, AB-P-R 001, China), and anti-TM9SF1 (PA5–84406, Invitrogen, USA).

### 2.9 Statistical analysis

Continuous data are presented as means ± standard deviation, while qualitative data are presented as N (percentage), and these data types were respectively compared with Student’s t-tests or chi-square tests, with one-way ANOVAs being used to compare 3 or more groups. Linear correlation analyses were used to assess correlations between *TM9SF1* expression and cytokines. The association between *TM9SF1* levels and ARDS severity was examined through univariate and multivariate logistic regression analyses in which odds ratios (ORs) and 95% confidence intervals (CIs) were calculated. The composite outcome of disease progression and death was analyzed with Cox regression analyses, calculating hazard ratios (HRs) and corresponding 95% CIs. Analyses were adjusted for demographic characteristics including age, sex, smoking status, drinking status, and history of disease.

The predictive performance of *TM9SF1*, other key indicators, and developed predictive models as tools for assessing ARDS severity and mortality was evaluated using receiver operating characteristic (ROC) curves. Predictive models for ARDS severity and mortality were developed through a multivariate analysis of those variables significant in univariate logistic regression analyses (*P* < 0.05), incorporating all variables that remained significant (*P* < 0.05) after adjustment for a range of covariates. Multivariate analysis results were also used to construct a nomogram related to ARDS severity. Nomogram performance was evaluated through discrimination analyses based on the concordance index and through the use of calibration curves and the Hosmer–Lemeshow calibration test, with bootstrapping (1,000x resampling).

The threshold for significance was a two-sided *P*-value < 0.05. Figures were constructed with GraphPad Prism 5 and R v3.6.3, while all statistical analyses were performed with SAS 9.4 software (SAS Institute, NC, USA).

## 3 Results

### 3.1 Study participant characteristics

Between November 1, 2022 and January 31, 2023, 239 total adult ARDS patients who were admitted to Xiangyang Central Hospital were enrolled in this study. Of these patients, 123 and 116 were respectively diagnosed with severe and nonsevere ARDS. ARDS patient demographic and clinical characteristics are presented in **Table 1**. Sex did not differ significantly between groups (female: 42.2% vs. 47.2%, *P* = 0.445), whereas the average age of severe ARDS patients was higher than that of individuals with nonsevere disease (73.67 ± 13.26 vs. 65.98 ± 17.03 , *P <* 0.001). Of these ARDS patients, 68.2% exhibited at least one comorbidity, among which hypertension (43.9%), diabetes mellitus (26.4%), and cardiovascular disease (18.0%) were the most common. Patients with severe disease were more likely than nonsevere patients to have hypertension (**Table 1**). The median duration of hospitalization for the overall ARDS patient cohort was 15 [8–23] days, with a significantly longer duration of hospitalization among individuals with severe disease as compared to those with nonsevere disease (19 [6–45] vs. 12 [8–18] days, *P* = 0.017).

**Table 1:** ARDS patient clinical and demographic characteristics classified according to disease severity.

Characteristics	Total (N=239)	Nonsevere (n=116)	Severe (n=123)	*P* value
Age, years	69.94 ± 26.87	65.98 ± 17.03	73.67 ± 13.26	<0.001
Sex				0.445
male	132 (55.2%)	67 (57.8%)	65 (52.8%)	
female	107 (44.8%)	49 (42.2%)	58 (47.2%)	
Primary cause of ARDS				0.978
pneumonia with COVID-19	80 (33.5%)	38 (32.8%)	42 (34.1%)	
pneumonia without COVID-19	68 (28.5%)	33 (28.4%)	35 (28.5%)	
aspiration	18 (7.5%)	8 (6.9%)	10 (8.1%)	
trauma	12 (5.0%)	5 (4.3%)	7 (5.7%)	
sepsis	50 (20.9%)	26 (22.4%)	24 (19.5%)	
others	11 (4.6%)	6 (5.2%)	5 (4.1%)	
Smoking history	38 (15.9%)	12 (10.3%)	26 (17.1%)	0.023
Drinking history	44 (18.4%)	15 (12.9%)	29 (23.6%)	0.034
Hypertension	105 (43.9%)	41 (35.3%)	64 (52.0%)	0.009
Diabetes mellitus	63 (26.4%)	25 (21.6%)	38 (30.9%)	0.101
Cardiovascular disease	43 (18.0%)	18 (15.5%)	25 (20.3%)	0.333
Stroke	27 (11.3%)	11 (9.5%)	16 (13.0%)	0.390
WBC (10^9^/L)	10.24 ± 5.63	9.25 ± 5.66	11.17 ± 5.46	0.008
NEU%	81.76 ± 13.27	77.98 ± 12.97	85.33 ± 12.62	<0.001
LYM%	11.17 ± 10.68	14.08 ± 9.97	8.42 ± 6.62	<0.001
MON%	6.28 ± 3.88	7.23 ± 3.76	5.39 ± 3.80	<0.001
Neutrophils (10^9^/L)	8.94 ± 6.09	7.64 ± 5.49	10.17 ± 6.36	0.001
Lymphocyte (10^9^/L)	0.84 ± 0.73	0.96 ± 0.53	0.72 ± 0.56	0.001
Monocytes (10^9^/L)	0.56 ± 0.40	0.57 ± 0.36	0.56 ± 0.44	0.848
HGB (g/L)	110.36 ± 25.89	113.29 ± 23.35	107.59 ± 27.63	0.087
HCT (%)	30.59 ± 7.84	36.11 ± 7.08	25.38 ± 8.40	<0.001
PLT (10^9^/L)	179.05 ± 111.35	198.95 ± 105.72	160.29 ± 114.85	0.007
PDW (fL)	14.11 ± 3.12	13.81 ± 3.03	14.40 ± 3.19	0.144
MPV (fL)	11.20 ± 1.31	11.01 ± 1.28	11.37 ± 1.33	0.169
PCT (%)	0.21 ± 0.11	0.22 ± 0.10	0.21 ± 0.12	0.486
CRP (mg/L)	55.83 ± 56.26	31.85 ± 25.57	78.45 ± 56.00	<0.001
ESR (mm/60min)	47.34 ± 28.52	38.42 ± 25.05	55.76 ± 32.05	<0.001
NLR	18.76 ± 4.40	13.02 ± 3.94	24.17 ± 7.93	<0.001
PLR	327.97 ± 54.77	264.28 ± 85.31	388.04 ± 48.00	<0.001
MLR	0.96 ± 0.16	0.79 ± 0.38	1.11 ± 0.50	<0.001
PT (s)	16.01 ± 6.92	12.86 ± 8.31	18.99 ± 5.70	<0.001
INR	1.27 ± 0.83	1.29 ± 1.13	1.26 ± 0.50	0.789
APTT (s)	36.37 ± 29.53	31.54 ± 10.72	40.92 ± 37.82	0.011
TT (s)	18.71 ± 13.93	19.77 ± 15.55	17.71 ± 5.03	0.165
D-dimer (mg/L)	5.25 ± 4.36	2.79 ± 1.88	7.57 ± 4.59	<0.001
PaO_2_ (mmHg)	72.43 ± 16.21	73.91 ± 20.08	71.03 ± 17.42	0.237
PaCO_2_ (mmHg)	52.29 ± 10.68	52.79 ± 11.03	51.81 ± 10.84	0.489
PaO_2_/FiO_2_ (mmHg)	236.84 ± 53.68	263.96 ± 52.78	211.27 ± 50.32	<0.001
SOFA	12 (9–16)	11 (7–14)	13 (8–17)	0.057
Hospital stay, days	15 (8–23)	12 (8–18)	19 (6–45)	0.017

Continuous variables are given as means ± standard deviation or medians (Q1-Q3) while categorical variables are presented as n (%). ARDS, acute respiratory distress syndrome; WBC, white blood cells; NEU%, neutrophil %; LYM%, lymphocyte %; MON%, monocyte %; HGB, hemoglobin; HCT, hematocrit; PLT, platelets; PDW, platelet distribution width; MPV, mean platelet volume; PCT, procalcitonin; CRP, C-reactive protein; ESR, erythrocyte sedimentation rate; NLR, neutrophil-lymphocyte ratio; MLR, monocyte lymphocyte ratio; PLR, platelet lymphocyte ratio; PT, prothrombin time; INR, international normalized ratio; APTT, activated partial thromboplastin time; TT, thrombin time; SOFA, Sequential Organ Failure Assessment.

Laboratory findings from patients grouped according to disease severity are shown in **Table 1**. Routine blood analyses revealed that patients with severe ARDS presented with higher WBCs (*P* = 0.008) and neutrophil counts (*P* = 0.001) together with reduced lymphocyte counts as compared to nonsevere patients (*P* = 0.001). HCT, lymphocyte %, monocyte %, neutrophil %, NLR, PLR, and MLR values also differed significantly among groups (all *P* values < 0.001). With respect to platelet and coagulation indices, individuals with severe disease presented with higher APTT, PT, and D-dimer levels (all *P* values < 0.05). CRP and ESR levels were also significantly elevated in individuals suffering from severe disease as compared to those with nonsevere disease (all *P*-values < 0.001). The PaO_2_/FiO_2_ ratio was also significantly reduced among severe patients (*P* < 0.001), while SOFA scores exhibited the opposite trend to those for the PaO_2_/FiO_2_ ratio.

All patients with ARDS completed the follow-up period through March 31, 2023, and were included in these analyses. Over the course of this follow-up interval, 64 (26.8%) patients died and 175 (73.2%) patients recovered. Similar differences in clinical and demographic characteristics were observed between patients in the severe ARDS group that died and recovered (**Table 2**). Relative to patients in the severe group that progressed, through who died exhibited several worse laboratory parameters including higher WBCs, neutrophil %, NLR, PLR, MLR, D-dimer, CRP, and SOFA score values together with decreases in lymphocyte %, monocyte %, and PaO_2_/FiO_2_ ratio (all *P*-values < 0.05).

**Table 2:** Demographic and clinical characteristics of ARDS patients with severe disease classified according to survival status.

Characteristics	Alive (n=74)	Dead (n=49)	*P* value
Age, years	68.57 ± 14.61	81.39 ± 48.85	0.036
Sex			<0.001
male	29 (39.2%)	36 (73.5%)	
female	45 (60.8%)	13 (26.5%)	
Smoking history	11 (14.9%)	15 (30.6%)	0.036
Drinking history	13 (17.6%)	16 (32.7%)	0.054
Hypertension	37 (50.0%)	27 (55.1%)	0.579
Diabetes mellitus	20 (27.0%)	18 (36.7%)	0.254
Cardiovascular disease	10 (13.51%)	15 (30.6%)	0.021
Stroke	7 (9.5%)	9 (18.4%)	0.150
WBC (10^9^/L)	10.52 ± 5.27	15.10 ± 5.65	<0.001
NEU%	83.33 ± 11.07	88.16 ± 14.17	0.036
LYM%	9.12 ± 5.68	7.02 ± 4.93	0.037
MON%	6.50 ± 4.10	3.83 ± 2.66	<0.001
Neutrophils (10^9^/L)	9.36 ± 6.99	11.90 ± 5.33	0.033
Lymphocyte (10^9^/L)	0.74 ± 0.51	0.58 ± 0.19	0.038
Monocytes (10^9^/L)	0.63 ± 0.44	0.47 ± 0.23	0.021
HGB (g/L)	108.34 ± 27.74	104.12 ± 27.59	0.409
HCT (%)	32.57 ± 8.24	32.10 ± 8.70	0.763
PLT (10^9^/L)	192.21 ± 132.71	138.57 ± 72.80	0.011
PDW (fL)	14.20 ± 3.41	14.70 ± 2.84	0.397
MPV (fL)	11.21 ± 1.32	11.60 ± 1.33	0.112
PCT (%)	0.22 ± 0.13	0.17 ± 0.07	0.015
CRP (mg/L)	68.98 ± 54.29	94.76 ± 56.06	0.012
ESR (mm/60min)	51.47 ± 34.01	57.67 ± 25.02	0.276
NLR	19.97 ± 12.61	30.23 ± 17.14	<0.001
PLR	334.22 ± 168.24	467.60 ± 121.30	<0.001
MLR	0.51 ± 0.20	1.10 ± 0.44	<0.001
PT (s)	13.98 ± 3.51	16.39 ± 7.61	0.019
INR	1.17 ± 0.34	1.38 ± 0.65	0.021
APTT (s)	36.34 ± 8.58	47.27 ± 27.26	0.002
TT (s)	17.75 ± 5.49	17.65 ± 4.38	0.915
D-dimer (mg/L)	4.89 ± 2.31	9.51 ± 4.10	<0.001
PaO_2_ (mmHg)	74.97 ± 20.13	70.02 ± 16.84	0.157
PaCO_2_ (mmHg)	54.24 ± 12.18	58.19 ± 9.24	0.056
PaO_2_/FiO_2_ (mmHg)	221.43 ± 37.68	203.19 ± 43.67	<0.001
SOFA	12 (6–15)	14 (9–19)	0.028
Hospital stay, days	17 (6–24)	22 (9–40)	0.012

Continuous variables are given as the mean ± standard deviation or median (Q1-Q3) while categorical data are presented as n (%). ARDS, acute respiratory distress syndrome; WBC, white blood cells; NEU%, neutrophil %; LYM%, lymphocyte %; MON%, monocyte %; HGB, hemoglobin; HCT, hematocrit; PLT, platelets; PDW, platelet distribution width; MPV, mean platelet volume; PCT, procalcitonin; CRP, C-reactive protein; ESR, erythrocyte sedimentation rate; NLR, neutrophil-lymphocyte ratio; MLR, monocyte lymphocyte ratio; PLR, platelet lymphocyte ratio; PT, prothrombin time; INR, international normalized ratio; APTT, activated partial thromboplastin time; TT, thrombin time; SOFA, Sequential Organ Failure Assessment.

### 3.2 Patients with severe ARDS exhibit increased TM9SF1 expression

**Figure 1:**
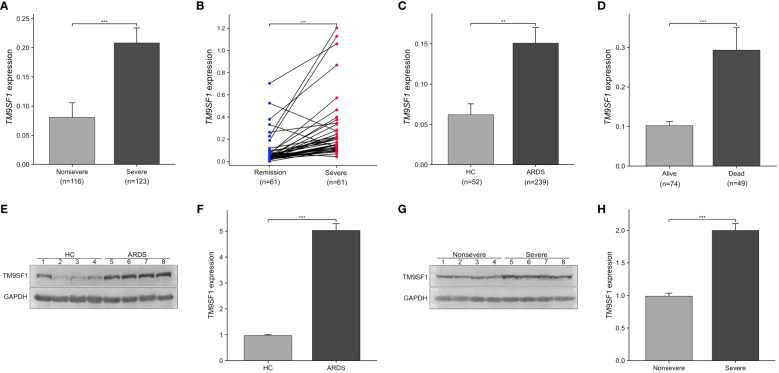
Severe ARDS patients exhibit elevated *TM9SF1* expression. **(A)** Patients with severe and nonsevere ARDS. **(B)** Paired samples from patients with ARDS, in the remission and severe groups. **(C)** Patients with ARDS and HCs. **(D)** Patients with severe ARDS that did or did not survive. **(E, F)** Comparison of TM9SF1 protein expression in PBMCs from patients with ARDS and healthy controls, assessed by western blotting followed by semi-quantification of band intensities using Image J software with GAPDH as the loading control. **(G, H)** TM9SF1 protein expression in PBMCs from patients with ARDS on transition from severe to non-severe disease, evaluated by western blotting and semi-quantification of band intensities using Image J software with GAPDH as the loading control. ARDS, acute respiratory distress syndrome; HC, health control. ^**^ *P* < 0.01; ^***^ *P* < 0.001.

Differential *TM9SF1* expression was assessed in ARDS patients and HCs (**Figure 1**). Blood samples from 61 ARDS patients were obtained during both periods of severe and stable remission, and samples were collected from 52 HCs at the same time. Significant increases in *TM9SF1* mRNA levels were detected among patients with severe ARDS relative to individuals with nonsevere disease (0.21 ± 0.03 vs. 0.08 ± 0.02, *P* < 0.001). When *TM9SF1* expression levels were analyzed in individuals during both severe disease and stable remission (61 pairs), *TM9SF1* expression was found to be significantly reduced with disease resolution (0.28 ± 0.04 vs. 0.10 ± 0.02, *P* < 0.001) (**Figure 1B**). There were also significantly higher *TM9SF1* expression levels in ARDS patients relative to HCs (0.15 ± 0.02 vs. 0.06 ± 0.01, *P* = 0.003) (**Figure 1C**). Independent-sample t-tests revealed that *TM9SF1* expression levels were also significantly higher among deceased ARDS patients as compared to surviving patients (0.29 ± 0.06 vs. 0.10 ± 0.01, *P* < 0.001) (**Figure 1D**).

In addition, we performed Western blotting to verify the TM9SF1 protein levels in PBMCs from 4 patients with ARDS and 4 healthy controls. It was found that the protein levels of TM9SF1 were upregulated 5.19-fold in patients with ARDS compared to healthy controls (**Figures 1E, F**). Additionally, TM9SF1 protein levels in PBMCs from patients with severe ARDS were also upregulated 2.02-fold compared to those from patients with non-severe ARDS (**Figures 1G, H**).

### 3.3 TM9SF1 expression is significantly correlated with cytokines in ARDS patients

**Figure 2:**
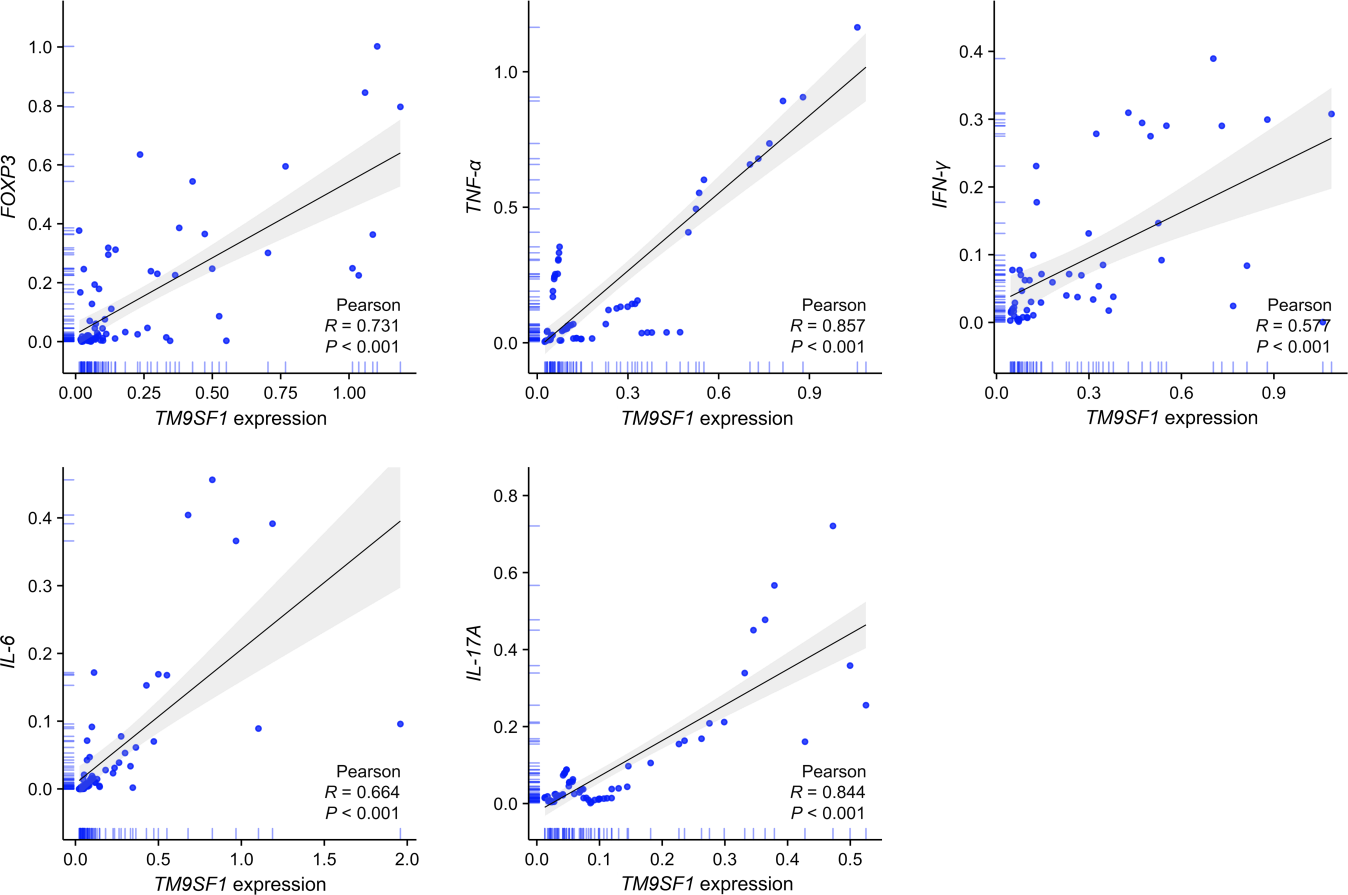
*TM9SF1* expression is significantly correlated with cytokine levels in ARDS patients. ARDS, acute respiratory distress syndrome; FOXP3, forkhead box P3; TNF-α, tumor necrosis factor-α; IFN-γ, interferon-γ; IL-6, interleukin-6; IL-17A, interleukin-17A. The shadows represent the 95% confidence intervals of the fitted line.

The release of excessively high cytokine levels is a hallmark of ARDS that can result in severe lung damage (17). To probe the relationship between *TM9SF1* mRNA levels and ARDS patient cytokine levels, correlation analyses were next conducted (**Figure 2**). This approach revealed that *TM9SF1* mRNA levels were significantly positively correlated with *interferon (IFN)-γ* (r = 0.577, *P* < 0.001) and *tumor necrosis factor (TNF)-α* (r = 0.857, *P* < 0.001) levels, with the same also being true for *interleukin (IL)-6* (r = 0.664, *P* < 0.001), *IL-17A* (r = 0.844, *P* < 0.001), and the *transcription factor forkhead box P3* (*FOXP3*) (r = 0.731, *P* < 0.001). When these same correlation analyses were only conducted in patients with severe ARDS, these correlations remained intact, with particularly strong correlations between the expression of *TM9SF1* and *IFN-γ* (r = 0.821, *P* < 0.001), *TNF-α* (r = 0.640, *P* < 0.001), and *IL-17A* (r = 0.613, *P* < 0.001). These data support a relationship between *TM9SF1* expression and plasma cytokines in ARDS patients, identifying this gene as a potential regulator of ARDS pathogenesis. The cytokine levels during remission and severe disease in patients with ARDS are presented in **Supplementary Figure S1**.

### 3.4 High levels of TM9SF1 expression may contribute to increased ARDS severity and mortality

The link between the expression of *TM9SF1* and ARDS severity was next probed through univariate and multivariate analyses (**Supplementary Table S2**). When these analyses were adjusted for patient demographic characteristics including age, sex, smoking status, drinking status, and history of disease, ARDS severity rose with increases in *TM9SF1* expression (OR = 2.43, 95% CI = 2.15–3.72, *P* = 0.005). When median *TM9SF1* expression levels were used to stratify patients into low- and high-risk groups, high-risk patients presented with a 3.03-fold increase in ARDS severity as compared to low-risk patients (OR = 4.03, 95% CI = 3.75–6.36, *P* < 0.001).

Cox regression analyses were further used to probe the link between the expression of *TM9SF1* and patient mortality (**Supplementary Table S3**). When these analyses were adjusted for demographic characteristics including age, sex, smoking status, drinking status, and history of disease, ARDS patient mortality risk rose as *TM9SF1* levels increased (HR = 2.27, 95% CI = 2.20–4.39, *P* = 0.001). Relative to patients with lower levels of *TM9SF1* expression, the survival time of patients expressing higher levels of this gene was reduced (HR = 3.10, 95% CI = 2.03–4.32, *P* = 0.008).

### 3.5 TM9SF1 can predict ARDS severity and mortality more effectively than other clinical indicators

ROC curve analyses were used to gauge the relative performance of *TM9SF1* and a range of other clinical indicators (WBCs, lymphocytes, lymphocyte %, monocytes, monocyte %, neutrophils, neutrophil %, NLR, MLR, PLR, CRP, D-dimer, PaO_2_/FiO_2_ and SOFA) when predicting ARDS severity (**Table 3**). Of the analyzed variables, *TM9SF1* exhibited the highest area under the ROC curve (AUC) when predicting disease severity (0.871, 95% CI = 0.785–0.924). The corresponding AUC values for MLR and D-dimer when predicting ARDS severity were 0.798 (95% CI = 0.645–0.847) and 0.811 (95% CI = 0.764–0.883). These results thus suggested that *TM9SF1* was the most efficacious marker for use when distinguishing between ARDS patients with severe and nonsevere disease. When using a *TM9SF1* expression cut-off value of 0.07, the respective sensitivity and specificity values were 87.3% and 84.7%.

**Table 3:** The predictive performance of *TM9SF1* and a range of other clinical indicators when predicting ARDS severity.

Indicators	AUC	95% CI	Sensitivity	Specificity
*TM9SF1*	0.871	0.785–0.924	0.873	0.847
WBC	0.733	0.658–0.808	0.703	0.657
Lymphocytes	0.715	0.613–0.756	0.747	0.784
Monocytes	0.730	0.695–0.823	0.719	0.685
Neutrophils	0.802	0.757–0.873	0.743	0.786
LYM%	0.712	0.643–0.781	0.736	0.628
MON%	0.748	0.674–0.821	0.795	0.738
NEU%	0.690	0.619–0.761	0.685	0.745
NLR	0.812	0.742–0.881	0.836	0.748
MLR	0.798	0.645–0.847	0.763	0.698
PLR	0.758	0.619–0.820	0.762	0.701
CRP	0.781	0.706–0.856	0.714	0.734
D-dimer	0.811	0.764–0.883	0.845	0.792
PaO_2_/FiO_2_	0.824	0.725–0.864	0.803	0.734
SOFA	0.791	0.701–0.842	0.788	0.742

ARDS, acute respiratory distress syndrome; AUC, area under the curve; CI, confidence interval; WBC, white blood cells; LYM%, lymphocyte %; MON%, monocyte %; NEU%, neutrophil %; NLR, neutrophil-lymphocyte ratio; MLR, monocyte lymphocyte ratio; PLR, platelet lymphocyte ratio; CRP, C-reactive protein; SOFA, Sequential Organ Failure Assessment.

Time-dependent ROC analyses were further used to probe the performance of *TM9SF1* and these other variables when predicting patient mortality (**Table 4**). In these analyses, *TM9SF1* levels again exhibited superior predictive utility with a C-index of 0.846 (95% CI = 0.752–0.917) for patient mortality, with corresponding sensitivity and specificity values of 87.5% and 82.6% at a cut-off value of 0.15. The respective C-index values for CRP, PaO_2_/FiO_2_, and SOFA scores when predicting ARDS patient mortality were 0.783 (95% CI = 0.646–0.905), 0.816 (95% CI = 0.719–0.853), and 0.797 (95% CI = 0.711–0.852). These results demonstrate that *TM9SF1* exhibits predictive performance better than that of a wide range of other clinical indicators when assessing ARDS patient disease severity and mortality risk. As such, *TM9SF1* may offer value as a novel biomarker to predict ARDS severity and patient survival.

**Table 4:** The predictive performance of *TM9SF1* and a range of other clinical indicators when assessing ARDS patient mortality through a time-dependent ROC analysis.

Indicators	Harrell's C-index	95% CI	Sensitivity	Specificity
*TM9SF1*	0.846	0.752–0.917	0.875	0.826
WBC	0.707	0.657–0.811	0.757	0.718
Lymphocytes	0.796	0.609–0.825	0.768	0.695
Monocytes	0.735	0.646–0.816	0.738	0.725
Neutrophils	0.758	0.659–0.823	0.816	0.755
LYM%	0.716	0.642–0.809	0.714	0.792
MON%	0.739	0.665–0.846	0.752	0.688
NEU%	0.759	0.668–0.813	0.733	0.704
NLR	0.804	0.701–0.911	0.831	0.795
MLR	0.758	0.623–0.895	0.756	0.711
PLR	0.799	0.688–0.924	0.796	0.707
CRP	0.783	0.646–0.905	0.765	0.799
D-dimer	0.806	0.693–0.889	0.813	0.728
PaO_2_/FiO_2_	0.816	0.719–0.853	0.794	0.757
SOFA	0.797	0.711–0.852	0.793	0.772

ARDS, acute respiratory distress syndrome; ROC, receiver operating characteristic; CI, confidence interval; WBC, white blood cells; LYM%, lymphocyte %; MON%, monocyte %; NEU%, neutrophil %; NLR, neutrophil-lymphocyte ratio; MLR, monocyte lymphocyte ratio; PLR, platelet lymphocyte ratio; CRP, C-reactive protein; SOFA, Sequential Organ Failure Assessment.

### 3.6 Nomogram development and validation

Multivariate logistic regression analyses incorporating all results that were significantly associated with ARDS severity in univariate analyses (*P* < 0.05) were next conducted to develop a predictive model. Those variables that were significant in this model following adjustment for covariates (*P* < 0.05) were considered independently associated with ARDS severity, and included *TM9SF1* levels, age, D-dimer, and CRP levels, with respective ORs of 2.31 (95% CI = 1.12–4.67, *P* = 0.017), 1.07 (95% CI = 1.02–1.11, *P* = 0.024), 1.48 (95% CI = 1.31–2.05, *P* = 0.018), and 1.02 (95% CI = 1.01–1.05, *P* = 0.035).

Multivariable Cox proportional hazards regression analyses similarly identified elevated *TM9SF1* expression (HR = 2.47, 95% CI = 2.18–5.49, *P* = 0.011), older age (HR = 1.36, 95% CI 1.22–4.19, *P* = 0.038), elevated NLR levels (HR = 1.81, 95% CI 1.01–3.25, *P* = 0.048), and elevated D-dimer levels (HR = 1.51, 95% CI = 1.08–2.12, *P* = 0.017) as being independently associated with the risk of mortality among patients with severe ARDS.

**Figure 3:**
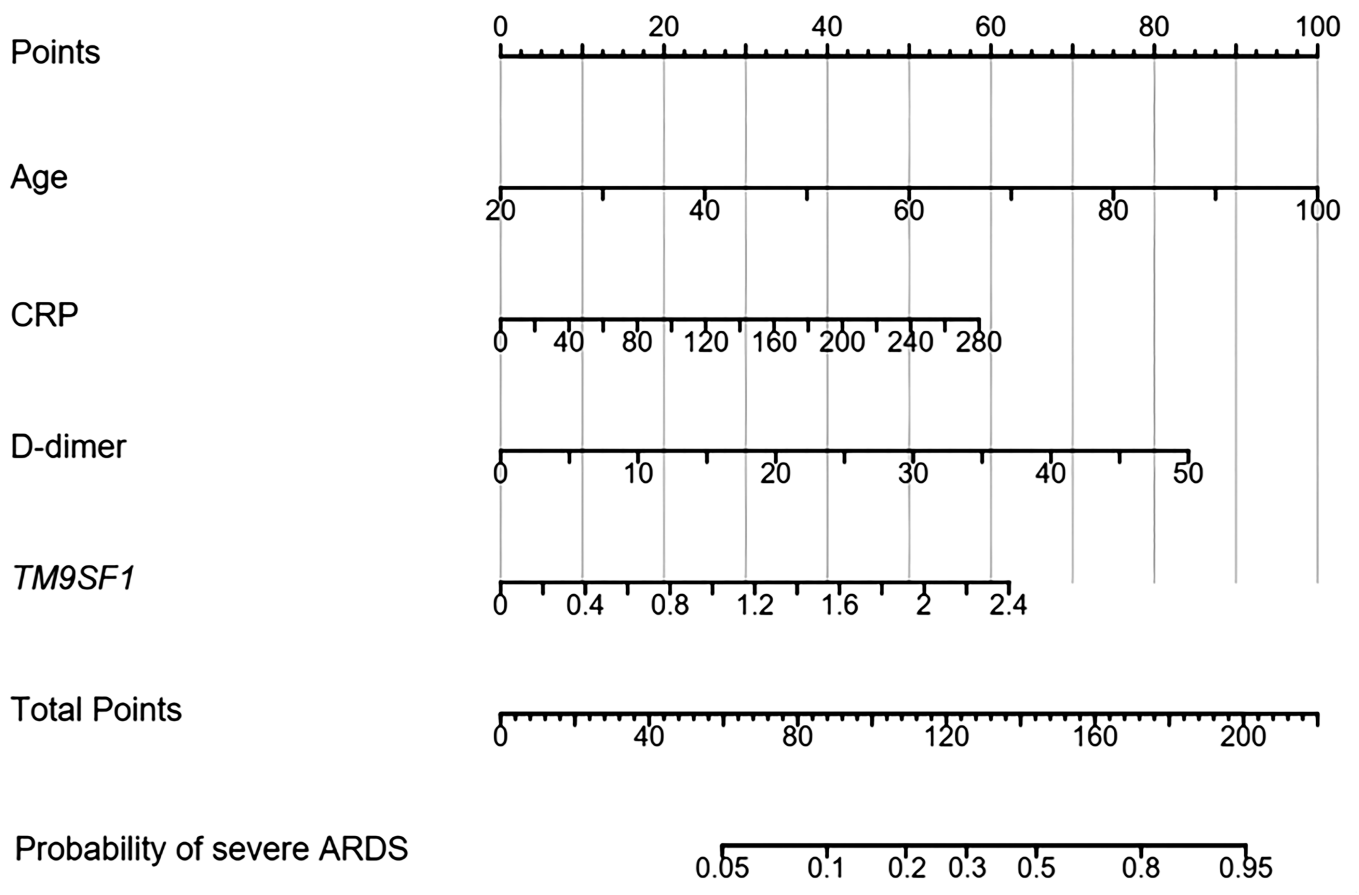
Nomogram developed to predict ARDS severity. ARDS, acute respiratory distress syndrome; CRP, C-reactive protein. Points: the individual scores corresponding to each predictor in the model in different groups/values. Total Points: the total score of each score corresponding to the values of all predictors. Probability: the predicted probability of event occurrence at the corresponding total point value.

**Figure 4:**
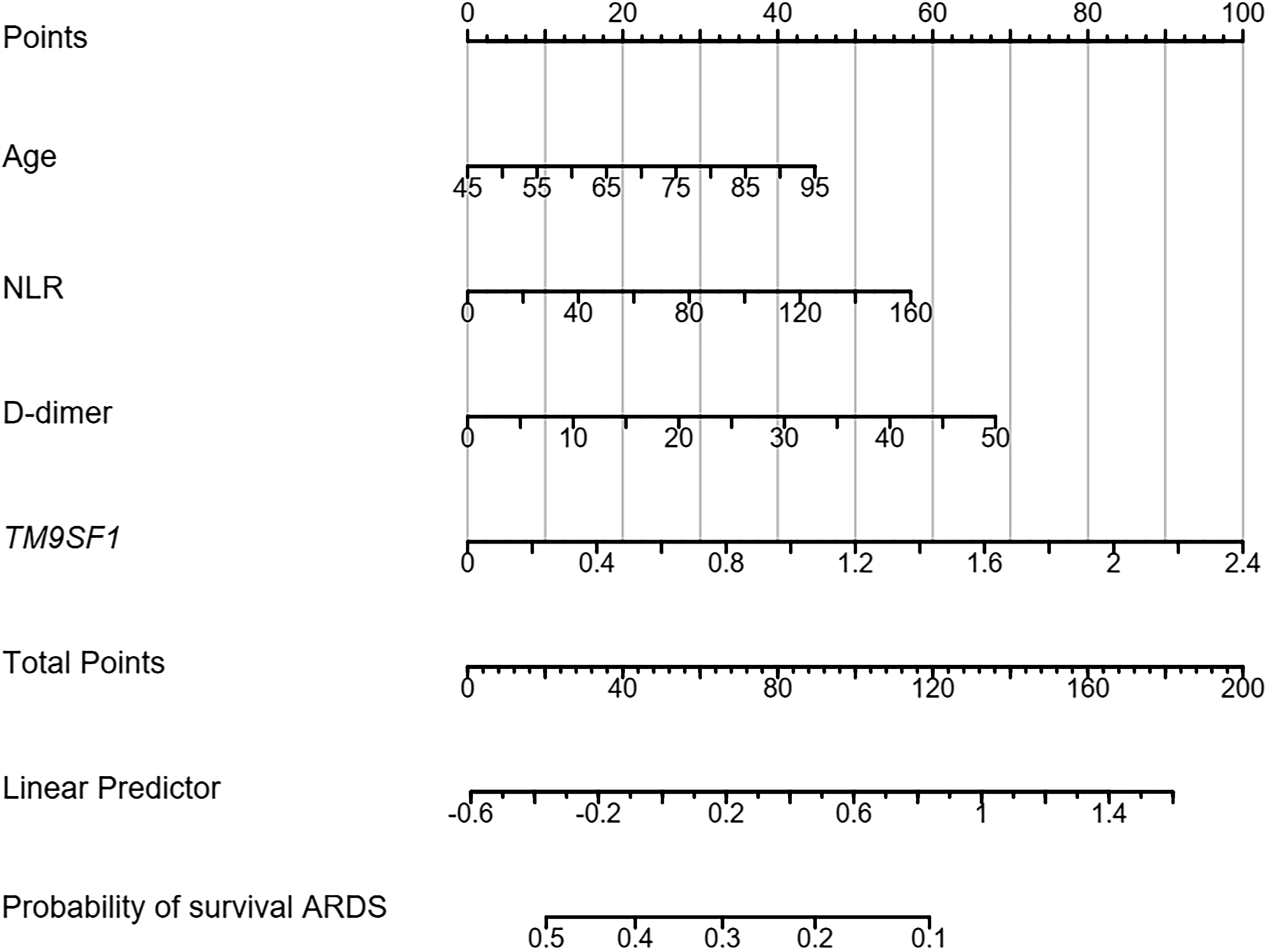
Nomogram developed to predict ARDS patient mortality risk. ARDS, acute respiratory distress syndrome; CRP, C-reactive protein. Points: the individual scores corresponding to each predictor in the model in different groups/values. Total Points: the total score of each score corresponding to the values of all predictors. Probability: the predicted probability of event occurrence at the corresponding total point value.

A nomogram for use in predicting severe ARDS development was established incorporating *TM9SF1* levels, age, CRP levels, and D-dimer levels based on the above multivariate analysis results (**Figure 3**). The odds of developing severe ARDS were computed for individual patients by summing the scores for each of these individual risk factors. Similarly, a predictive nomogram for severe ARDS patient mortality risk was developed incorporating *TM9SF1* expression, age, NLR, and D-dimer levels (**Figure 4**).

ROC curves were used to validate the nomogram designed to predict ARDS severity, yielding an AUC of 0.887 (95% CI = 0.715–0.943), a sensitivity of 87.9%, and a specificity of 86.8%. The validation of the nomogram used when predicting severe ARDS patient mortality yielded a C-index of 0.890 (95% CI = 0.627–0.957). Patients were separated into low- and high-risk groups based on total points to evaluate prognostic outcomes, revealing an optimal risk stratification value of 112.4 points.

## 4 Discussion

This prospective cohort study explored the utility of *TM9SF1* as an early predictor of ARDS severity and patient survival outcomes. Severe ARDS patients presented with significantly higher *TM9SF1* expression as compared to those with nonsevere disease and HCs. Significantly higher *TM9SF1* mRNA levels were also detected in patients who experienced fatal ARDS as compared to those who did not. Multivariate logistic regression approaches indicated that increases in *TM9SF1* levels were associated with greater disease severity, and higher *TM9SF1* expression was also associated with greater patient mortality risk in Cox regression analysis. ROC curves revealed that the predictive performance of *TM9SF1* was superior to that of many other clinical indicators associated with ARDS severity and patient mortality. Predictive models incorporating *TM9SF1* expression levels were also established to assess ARDS severity and mortality risk, and these models exhibited satisfactory predictive performance. Preliminary investigation focused on the biological link between *TM9SF1* and immune regulation in ARDS patients further revealed that *TM9SF1* mRNA levels were strongly correlated with plasma cytokines. These findings suggest that *TM9SF1* may offer utility as a novel biomarker for the personalized assessment of ARDS patient disease status and prognosis. In addition, the findings of the study provide a scientific basis for the individualized treatment of ARDS.

Many ARDS-related biomarkers and clinical parameters have been described to date (18–22). For example, Medford et al. explored polymorphisms in the *VEGF* gene and determined that patients with the T allele for a *VEGF* polymorphism exhibited greater ARDS incidence (18). Tejera et al. similarly noted that the rs324420-FAAH polymorphism was strongly correlated with ARDS (19). Many studies have also revealed that NLR, PLR, and/or MLR values can offer excellent utility as a predictor of ARDS clinical course and the risk of mortality among ARDS patients (23, 24). In a separate multicenter study, risk factors associated with ARDS severity were found to include age, sex, PaO_2_/FiO_2_ ratio, and SOFA scores (25). While these studies probed the predictive performance of individual biomarkers when assessing ARDS severity and/or mortality risks, none constructed multi-factor predictive models or evaluated both clinical and epigenetic biomarkers in order to simultaneously consider the effects of these various factors in a comprehensive manner. Here, *TM9SF1* levels were found to outperform a range of clinical indicators with respect to their ability to predict ARDS severity and affected patient mortality. When predictive models incorporating *TM9SF1* were established, they exhibited satisfactory predictive performance for ARDS severity and patient prognostic outcomes, outperforming any predictive models reported in these prior studies.

TM9SF family proteins are highly conserved across species, and consist of 9 putative transmembrane domains together with a large non-cytoplasmic domain (26, 27). In mammals, four members of this protein family (TM9SF1–4) have thus far been identified (26), with a limited number of prior reports having documented the relationships between these proteins and certain cancers (28–31). TM9SF1 is ubiquitously expressed at the protein level in many human tissues, and it shares 27–31% sequence identity with its yeast counterpart, suggesting that it likely plays important intracellular functions (32). While there have been prior analyses focused on patterns of TM9SF1 expression (33, 34), its functional characteristics remain poorly understood. Work from our group recently unveiled an increase in TM9SF1 expression levels in the lungs of mice suffering from LPS-induced acute lung injury, while *TM9SF1* knockdown in these animals was found to alleviate the pulmonary inflammation induced by LPS through the enhancement of autophagic activity (12).

Elevated levels of many cytokines such as *IL-6*, *IL-17A*, *TNF-α*, and *IFN-γ* and the onset, severity, and prognosis of ARDS have long been documented (17). Elevated *IL-6* expression has been observed among patients at risk of ARDS or with established disease, with particularly high levels among non-survivors (35–38). The IL-17 cytokine family consists of six proteins (IL-17A/B/C/D/E/F), and *IL-17A* levels have previously been shown to be positively correlated with various measures of lung function, inflammation, and disease severity in ARDS patients (39, 40). The production of TNF-α is central to acute inflammatory processes and it is enhanced in ARDS patients, individuals with severe COVID-19, and individuals with other forms of severe pneumonia (35, 36, 38). As the only type II IFN protein, IFN-γ serves as a central regulator of innate and adaptive immune function, although its relationship with ARDS has only recently been explored. For example, one recent study included IFN-γ in a panel of plasma biomarkers used to define a “reactive” disease subtype associated with higher rates of mortality in the ICU (41). Analyses of ARDS cases associated with SARS-CoV-2, bacterial pneumonia, human adenovirus (HAdV), and IAV/H1N1 have also documented significant increases in levels of IFN-γ and multiple other cytokines as compared to HCs (42). Notably, more pronounced increases in IFN-γ levels were observed in patients with HAdV infection who developed ARDS relative to patients who did not develop this complication, and a negative correlation was documented between the levels of this cytokine and the PaO_2_/FiO_2_ ratio (42). FOXP3 is an important transcription factor that controls the functions of regulatory T cells (Tregs) (43). Some reports have suggested that ARDS patients exhibit increases in Treg percentages of FOXP3 expression (43), whereas FOXP3 levels reportedly return to normal levels in convalescent or recovering patients (44). In another recent report, increases in CD25+ CD127+ FOXP3+ Tregs were observed together with the enhancement of the suppressive activity of these cells in individuals with severe COVID-19 (45). Bronchoalveolar lavage fluid samples from COVID-19 patients with ARDs have been shown to exhibit increases in FOXP3+Tregs and Th17 cells together with overall reductions in T cell numbers (46). Our group previously demonstrated that LPS-induced lung injury was associated with TM9SF1 upregulation at the mRNA and protein levels, while knocking down its expression was sufficient to protect against lung inflammation and to inhibit the production of cytokines including sTNF-α. In the present clinical study, a positive correlation was detected between *TM9SF1* expression and levels of cytokines including IL-6, IL-17A, TNF-α, and IFN-γ as well as increases in FOXP3 levels, offering further support for the link between elevated *TM9SF1* levels and greater ARDS severity. However, the precise mechanisms that link *TM9SF1* and inflammatory immune regulation in patients with ARDS remain uncertain and warrant future investigation.

Here, *TM9SF1* expression levels in PBMCs from ARDS patients were found, for the first time, to be significantly elevated. This, coupled with the observed positive correlation between *TM9SF1* expression and ARDS severity, offered further support for the involvement of TM9SF1 in the incidence of pulmonary inflammation (12). We also performed Western blotting to verify the TM9SF1 protein levels in PBMCs. It was found that the protein levels of TM9SF1 were upregulated 5.19-fold in patients with ARDS compared to healthy controls. Additionally, TM9SF1 protein levels in PBMCs from patients with severe ARDS were also upregulated 2.02-fold compared to those from patients with non-severe ARDS. TM9SF1 has also been linked to a range of other conditions, including certain tumors (47, 48), ruptured intracranial aneurysms (34), and varicose spermatic veins (49). First identified as a marker associated with autophagy (50), TM9SF1 has since been confirmed to play roles in autophagic activity and regulating inflammatory factors (12). The precise molecular mechanisms whereby TM9SF1 functions, however, remain poorly understood. Efforts to clarify the functions of TM9SF1 are further hampered by the complex structure of this protein with its nine transmembrane domains. As such, while our team previously documented a role for this protein as a regulator of autophagy that controls lung inflammation in a mouse model system (12), the precise mechanisms linking TM9SF1 to inflammatory activity await further characterization.

This study is the first to have explored the functions or documented the performance of *TM9SF1* as an early predictor of disease severity and mortality in ARDS patients. The novel predictive models and associated nomograms incorporating *TM9SF1* designed herein were able to effectively predict ARDS patient status and clinical outcomes. However, this study is subject to some limitations. For one, this study only enrolled patients from a single hospital. As the overall patient cohort size was large and these patients were from many cities throughout China, however, these results are likely to be more representative and robust. In the future, a multicenter study will be performed to validate the present conclusions. Secondly, the average PaO_2_/FiO_2_ levels in patients with severe ARDS were not that low. In terms of the criteria by which the severe group was defined, the high PaO_2_/FiO_2_ level was apparent mainly in patients without invasive mechanical ventilation. When these patients were treated with analgesic sedation and high PEEP and were mechanically ventilated, the detected PaO_2_/FiO_2_ levels were higher than those in patients with invasive mechanical ventilation. As a result, the overall average PaO_2_/FiO_2_ levels in patients with severe ARDS were slightly higher. In addition, further research will be vital to clarify the immunological mechanisms whereby *TM9SF1* exerts its effects on the pathogenesis of ARDS.

## 5 Conclusions

In summary, the present analyses revealed significantly increased *TM9SF1* mRNA expression in patients with severe ARDS, and ARDS severity rose with increasing *TM9SF1* expression. In addition, high *TM9SF1* levels were associated with a greater risk of mortality among those ARDS patients with severe disease. *TM9SF1* outperformed a range of other clinical indicators with respect to its ability to predict ARDS patient mortality and disease severity, and predictive models incorporating *TM9SF1* were ultimately developed and found to exhibit satisfactory performance as a means of assessing ARDS severity and patient mortality risk. Based on these findings, *TM9SF1* can serve as a novel biomarker for the individualized assessment of ARDS severity and patient prognosis in individuals suffering from ARDS.

## Data availability statement

The raw data supporting the conclusions of this article will be made available by the authors, without undue reservation.

## Ethics statement

The studies involving humans were approved by the Ethics Committee of Hubei University of Arts and Science. The studies were conducted in accordance with the local legislation and institutional requirements. The participants provided their written informed consent to participate in this study.

## Author contributions

FC: Data curation, Investigation, Methodology, Writing – original draft. LZ: Conceptualization, Data curation, Formal Analysis, Methodology, Writing – original draft. ZZ: Data curation, Investigation, Methodology, Validation, Writing – review & editing. XS: Data curation, Formal Analysis, Investigation, Validation, Writing – review & editing. JiX: Data curation, Investigation, Methodology, Resources, Writing – review & editing. ZY: Data curation, Investigation, Methodology, Resources, Writing – review & editing. BG: Data curation, Investigation, Methodology, Validation, Writing – review & editing. ML: Data curation, Investigation, Methodology, Software, Validation, Writing – review & editing. HC: Data curation, Investigation, Methodology, Validation, Writing – review & editing. HX: Conceptualization, Data curation, Investigation, Resources, Validation, Writing – review & editing. MH: Data curation, Investigation, Methodology, Visualization, Writing – review & editing. YL: Data curation, Formal Analysis, Investigation, Methodology, Writing – review & editing. GQ: Formal Analysis, Investigation, Methodology, Software, Writing – review & editing. KW: Data curation, Funding acquisition, Methodology, Resources, Software, Validation, Writing – review & editing. FZ: Data curation, Methodology, Resources, Software, Validation, Writing – review & editing. JuX: Investigation, Software, Validation, Visualization, Writing – review & editing, Funding acquisition.
