# Supplementary material for: TM9SF1 offers utility as an efficient predictor of clinical severity and mortality among acute respiratory distress syndrome patients
Source: Front Immunol. 2024 Jun 3;15:1408406. doi: 10.3389/fimmu.2024.1408406 (PMC11180774; doi:10.3389/fimmu.2024.1408406)
Supplement: Supplementary file 1 [file DataSheet_1.pdf]

Table S1 RT-PCR primer sequences in this study

Primer name	Sequence (5'-3')	Organism
GAPDH-F	GGAGCGAGATCCCTCCAAAAT	human
GAPDH-R	GGCTGTTGTCATACTTCTCATGG	human
TM9SF1-F	GCACCCTGTCGCACCAAG	human
TM9SF1-R	GACAAAGAAGAGGATGCCGTAC	human
TNF-α-F	GACAGATGTGGGGTGTGAGAA	human
TNF-α-R	TCTGTGTGCCAGACACCCTA	human
IFN-γ-F	TCCAGTTACTGCCGGTTTGA	human
IFN-γ-R	TGGAAGCACCAGGCATGAAA	human
IL-6-F	GTCCAGTTGCCTTCTCCCTG	human
IL-6-R	CTGAGATGCCGTCGAGGATG	human
IL-17A-F	TAATGGCCCTGAGGAATGGC	human
IL-17A-R	AGGAAGCCTGAGTCTAGGGG	human
FOXP3-F	TCTTCCTTGAACCCCATGCC	human
FOXP3-R	AAATGTGGCCTGTCCTGGAG	human

Table S2 The association between *TM9SF1* expression and the severity of ARDS

	**OR (95% CI)[a]**	**OR (95% CI)[b]**
***TM9SF1* level**	2.48 (2.11–3.53)***	2.43 (2.15–3.72)**
**Subgroup**		
low level	Reference	Reference
high level	4.11 (3.57–6.35)***	4.03 (3.75–6.36)***

** P<0.01, *** P<0.001.

[a] Univariate analysis.

[b] Adjusted for age, sex, smoking status, drinking status and history of disease.

ARDS, acute respiratory distress syndrome; OR, odds ratio; CI, confidence interval.

Table S3 The association between *TM9SF1* expression and the prognosis of ARDS

	**HR (95% CI)[a]**	**HR (95% CI)[b]**
***TM9SF1* level**	2.31 (2.12–4.34)***	2.27 (2.20–4.39)**
**Subgroup**		
low level	Reference	Reference
high level	3.19 (2.06–4.49)**	3.10 (2.03–4.32)**

**P<0.01, ***P<0.001.

[a] Univariate analysis.

[b] Adjusted for age, sex, smoking status, drinking status and history of disease.

ARDS, acute respiratory distress syndrome; HR, hazard ratio; CI, confidence interval.

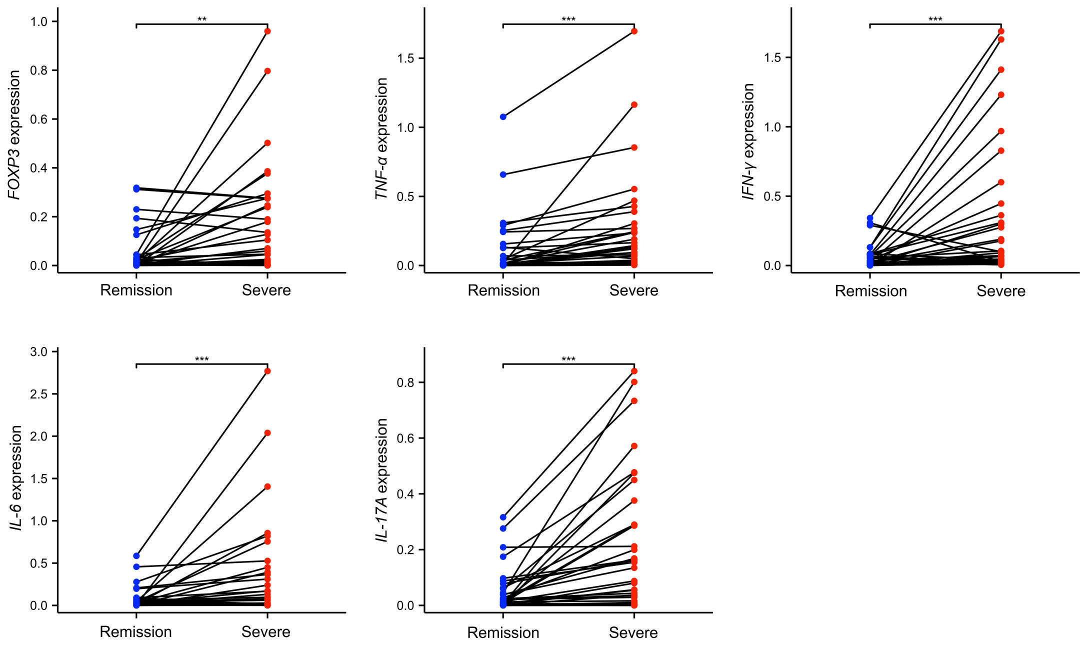

Figure S1 Comparison of cytokine levels in the remission and severe status of patients with ARDS. ARDS, acute respiratory distress syndrome; FOXP3, forkhead box P3; TNF-α, tumor necrosis factor-α; IFN-γ, interferon-γ; IL-6, interleukin-6; IL-17A, interleukin-17A. $^{**}P < 0.01$; $^{***}P < 0.001$.

Figure S1 description: Cytokine levels were compared between the severe and remission status of patients with ARDS. Figure S1 shows that when the condition of patients with ARDS gradually improved and the disease status changed from severe to remission, the expression level of *FOXP3* decreased concurrently; the difference was statistically significant (0.15 ± 0.04 vs. 0.05 ± 0.01, P = 0.005). The difference analysis of other cytokines, including *TNF-α*, *IFN-γ*, *IL-6*, and *IL-17A*, also showed similar results.
